# Editorial: Nanotechnologies in Neuroscience and Neuroengineering

**DOI:** 10.3389/fnins.2020.00033

**Published:** 2020-02-12

**Authors:** Ioan Opris, Mikhail A. Lebedev, Victor Manuel Pulgar, Ruxandra Vidu, Marius Enachescu, Manuel F. Casanova

**Affiliations:** ^1^Department of Biomedical Engineering, University of Miami, Coral Gables, FL, United States; ^2^Department of Neurobiology, Duke University, Durham, NC, United States; ^3^Center for Bioelectric Interfaces of the Institute for Cognitive Neuroscience, National Research University Higher School of Economics, Moscow, Russia; ^4^Department of Information and Internet Technologies of Digital Health Institute, I.M. Sechenov First Moscow State Medical University, Moscow, Russia; ^5^Department of Pharmaceutical Sciences, Campbell University, Buies Creek, NC, United States; ^6^Department of Obstetrics and Gynecology, Wake Forest School of Medicine, Winston-Salem, NC, United States; ^7^Department of Chemical Engineering and Materials Science, University of California, Davis, Davis, CA, United States; ^8^Center of Surface Science and Nanotechnology, Politehnica University of Bucharest, Bucharest, Romania; ^9^Department of Biomedical Sciences, University of South Carolina School of Medicine at Greenville, Greenville, SC, United States; ^10^Department of Pediatrics, Greenville Health System, Greenville, SC, United States

**Keywords:** nanotechnology, neuroengineering, nanoparticles, magnetic tunneling junctions, biosensors, multielectrode arrays, memistor, brain machine interfaces

Neuroscience and Neuroengineering operate at the cellular level and their association with Nanotechnology is bringing unexpected strides. During the last decade, we have witnessed an unprecedented increase in the successful application of Nanotechnology to both basic Neuroscience and to Clinical Practice. Novel nanotechnologies are expected to bring important insights on brain mechanisms and medical care to patients. The topic theme, “Nanotechnologies in Neuroscience and Neuroengineeringy” details attempts from different fields to improve brain performance in both healthy people and to patients suffering from neurological disabilities. Twenty-seven articles contributed by 122 authors composed this Research Topic on various aspects of Nanotechnologies applied in Neuroscience and Neuroengineering.

## Nanotechnologies in Neuroscience

Among the nanotechnologies that emerged recently in neuroscience, we cover the nanoparticles (including magnetic nanoparticles) and their involvement in therapy, the blood-brain-barrier, the nano-electrical and chemical stimulation, as well as recent insights into neuro-engineering, involving the characterization of biophysical features of neural cells and the function of neural microcircuits. Finally, we briefly discuss aspects of neural interfacing that have already been confirmed as feasible for brain machine interfaces and sensors.

### Nanoparticles

Nanoparticles are ultrafine units in the microscopic field of few to hundreds of nanometers, but less than a micron in size. Nanomagnetic and charged particles are endowed with valuable interactive abilities for neuronal cells. Pinkernelle et al., assessed the “bio-functionality” of growth factors using appropriate biological models. Thus, successful “functionalization” of magnetic nanoparticles with growth factors seems dependent on their “binding” chemistry. These magnetic nanoparticles support regeneration within the nervous system. Amirav et al., reviewed some recent magneto-fluorescent markers and highlighted key differences between them, in terms of durability and relevant approaches. They focused on the intracellular labeling potential and basic functional sensing MRI, with assays that enable the imaging of cells at microscopic and mesoscopic scales. Also, the limitations of available imaging markers have been reviewed/discussed while keeping in mind the possibility of *in vivo* neural imaging and large-scale brain mapping. Infante, proposed to examine the affinity and nanoparticle-based strategies for the delivery of neurotrophic factors to the spinal cord in an adequate, tunable, and safe therapeutic manner.

### Magnetic Nanoparticles and Magnetic Tunneling Junctions

Cellular processes such as the deformation of membrane, the transport of organelles or the migration of cells are sensitive to mechanical forces, operating through the “chaperoning” force-inducing nanoparticles in electrical/magnetic field gradients, with spatial precision in the range of sub-micrometers. Gahl and Kunze used force-mediating magnetic nanoparticles to generate neuronal cell function. Moretti et al., produced the first bio-magnetic chip using a novel technology based on magnetic tunnel junction (MTJ) for cell culture, and demonstrated how these sensors are biocompatible. Such advancements of nano-magnetic field in cellular organization/communication/signaling and intracellular trafficking can be used in the next generation of neurotherapeutic devices.

### Therapeutic Approaches

A series of therapeutically novel approaches emerged and were discussed in our Research Topic due to their potential applications. We mention here the ones involving: (i) carbon nanomaterials, such as nanotubes, graphene, nano-onions, or fullerenes for therapy, (ii) biosensing and imaging approaches that have antioxidant action, (iii) intrinsic photoluminescence, (iv) their ability to cross the BBB, carry oligonucleotides and cells, and (v) to induce cell differentiation. Fernandes et al., used liposomes and carbon nanomaterials in recent diagnosis and therapies in acute ischemic stroke. Liposomes represent a biomimetic system, with composition, structural organization, and properties very similar to biological membranes. Carbon nanomaterials, not being naturally parts of the human body, reveal new modes of interaction and integration with biological molecules and systems, resulting in completely unique pharmacological properties.

Novel genetic neuroprotective cell therapeutics are bringing promising approaches for the regenerative functions of the eye. Nafissi and Foldvari discussed these genetic nanotechnology neuroprotective therapies in glaucoma. The development of highly specific gene delivery methods which are safe and non-invasive are of crucial importance in ophthalmology. Nadeau reported the initial photophysical characterization of a new genetically encoded voltage sensor (based upon the fluorescence of rhodopsins), namely the “proteorhodopsin optical proton sensor” (PROPS). This is the first sensor capable of indicating the changes in membrane voltage by means of changes in fluorescence. Nadeau reported in two strains of *Escherichia coli*, a nanosecond time-resolved emission of this protein, before and after membrane depolarization.

d'Amora and Giordani have shown that zebrafish is a good animal model for “high-throughput” screening of chemicals, because of their small size, low-price, and transparency. Zebrafish has emerged as a powerful tool for screening developmental neurotoxicity. Convertino et al. elaborate on the graphene‘s potential for nerve tissue regeneration hinting to novel approaches of active nerve conduits for peripheral neuron survival and outgrowth. Moldovan et al. carried out experiments in adult transgenic mice with fluorescent tagged liposomes that provided insight into the local anesthetic effect of nanomedicines in post-operative pain. The effect of local anesthetic nanomedicines has important implications for humans.

### Blood Brain Barrier

Restorative strategies of brain function after stroke are centered on the repairing of cerebral endothelial and parenchymal cells. Communication between the cells and signaling within the neurovascular unit, including the multicellular brain-vessel-blood interface, with its highly selective blood-brain barrier (BBB), are crucial to the homeostasis of the central nervous system. Zagrean et al.'s work highlights the important role of exosomes in mediating the crosstalk of the cells within the neurovascular unit. It further reveals the restorative therapeutic potential of exosomes in ischemic stroke, a frequent neurologic condition still in need of an effective therapy. Pulgar then discussed transcytosis across the BBB. Pulgar draws our attention to the physiological operation of “receptor-mediated transcytosis” (RMT) to carry load across the brain endothelial cells toward brain parenchyma, exemplifying critical advances in RMT-mediated brain drug delivery.

### Nano-Electrical and Chemical Stimulation

A generation of minimally invasive or non-invasive neural stimulation techniques is being developed, supported by nanotechnology to reach high spatial resolution. In these approaches of neural stimulation, as pointed out by Wang and Guo, a nanomaterial transforms a faraway transmitted primary stimulus (like a magnetic or ultrasonic signal), into a localized secondary stimulus, such as, an electric field in order to stimulate neurons. Stimulating neural systems with “applied” electric field (EF) are a common tool for testing network responses. Tang-Schomer et al. used a “gold wire-embedded silk protein film-based interface” culture to examine the effects of “applied” EFs on neuronal networks in *in vitro* cultures. Cortical cultures displayed large-scale oscillations, synchronized by EF at specified frequencies. These effects of EF on random neuronal networks have significant implications for studies of brain function and neuromodulation. Furthermore, Goldental et al. mimicking the collective firing patterns of connected neurons, which proved the emergence of cooperative phenomena like synchronous oscillations, the coexistence of fast γ and slow δ oscillations, and other dynamical phenomena within large-scale neuronal networks.

Novel nanofluidic mechanisms like hydrophobic gating, suggested by Jones and Stelzle, may support the control of chemical release appropriate for mimicking neurotransmission. Nanofluidic chemical release facilitates fast, high resolution neurotransmitter-based neurostimulation, that could bring improvements over electrical neurostimulation.

## Neuroengineering

Nanotechnology is a fast-developing field, that provides simple and efficient tools to study the brain in health and disease. Of particular importance are biosensors, multi-electrode arrays, memory resistive devices, and brain machine interfaces.

### Biosensors

Hossain et al. reported the design and implementation of a “GABA microarray probe.” The probe consists of two distinct micro-biosensors, one for glutamate (Glu) and the other for GABA detection, modified with Glu oxidase and GABASE enzymes, respectively. The neurotransmitters GABA and Glu may be detected in real time, simultaneously/continuously, both: *in vitro* and *ex vivo*. The detection of GABA by such probe is based upon the “*in-situ* generation of α-ketoglutarate” from the oxidation that occurs at the Glu micro-biosensor. The GABA probe has been successfully tested in a slice preparation from a rat brain. These results show that the developed GABA probe represents a novel and valuable neuroscientific tool that could be utilized in studies of brain disorders involving the combined role of GABA and Glu signaling.

Many challenges of sensor development, including the bioengineered probes and sensors, arise when the physiological and pathological biomarkers are tested in neural cells (Maysinger et al.). The nanoparticle-based sensors have the ability to detect properties (biochemical and physiological) of neurons and glia, and to generate signals proportional to the changes (physical, chemical, or electrical) in these cells (Maysinger et al.). Among the most used nanostructures are the carbon-based structures (such as C-dots, graphene, and nano-diamonds), the quantum dots (QDs), and the gold nanoparticles. They are capable to detect/measure activity of proteases (metalloproteinases, caspases), ions, and other biomolecules under physiological or pathological conditions in neuronal cells. Such genetically manipulated probes and sensors are useful to reveal the changes in protease activities or calcium ion concentrations.

Moretti et al. demonstrated the biocompatibility of a magnetic sensor array for the detection of neuronal signals in the *in vitro* culture.

### Multielectrode Arrays (MEA)

MEA has been developed and used extensively in basic and applied research in neuronal- and cardiomyocyte-networks, both *in vivo* and *in vitro* (Spira et al.). The MEA platforms consisting of thousands of sensors (with high-density, small diameter, and low impedance), use vertical nanowires that pass through the cultured cell's membrane and record the action potentials in a similar manner to that of a sharp intracellular microelectrode. Spira's team developed a bioinspired approach in-which cell's energetic resources are utilized with extracellular gold microelectrodes to record attenuated synaptic- and action-potentials with characteristic features resembling those of intracellular recordings. Moreover, the approach allowed to record intracellular potentials by an array of extracellular electrodes.

Intracortical microelectrodes (IME) have been extensively used to study various functions of the nervous system. Recent strategies to enhance interfacing with the brain's systems have been suggested by methods that mimic the biological tissue. Kim et al. review focusses on nano-architecture, a concept that considers the surface of the implant. Different nano-architectural approaches have been discussed to enhance the “biocompatibility” of IMEs, increase the recording quality, and augment the longevity of the implant.

Microelectrode material together with cell culture medium play important roles in the health of a cell as derived from *in vitro* electrophysiological studies. Ryynänen et al. reported an “ion beam assisted e-beam deposition” (IBAD) based process as being an alternative to the titanium nitride (TiN) method of deposition for “sputtering” in the fabrication of “TiN microelectrode arrays” (MEAs). The developed IBAD TiN process enables the MEA manufacturers with more choices as to which method to use in order to deposit TiN electrodes. The medium evaluation results remind that in addition to electrode material the insulator layer and cell culturing medium keep a crucial role in successful long-term MEA measurements.

### Resistive Memory Devices

Resistive memory devices are a pioneering technology inspired by the brain mechanisms. Resistive random-access memory (RRAM) arrays use little energy and hold a potential for enormous densities. An interesting type of RRAM was demonstrated recently to have alternating (dynamic switching) current rectification properties, like those of CMOS transistors (Berco). Such artificial synaptic devices can be switched between two modes (excitatory and inhibitory) to double the array density and to significantly reduce the peripheral circuit complexity. Gavrilov et al. discusses next the “associative spatial-temporal memories” based on neuromorphic networks with restricted connectivity, termed-“CrossNets.” Such networks have the capability to be implemented naturally in nanoelectronic hardware with hybrid memristive circuits (a memistor is a nanoelectric element of circuitry used in parallel computing memory technology). This may allow extremely high energy efficiency, comparable to that of the biological cortical circuits, functioning at a much higher operation speed. Numerical simulations performed by Gavrilov et al. and confirmed with analytical calculations, show that the characteristics depend significantly on the method of information recording into the memory. Most importantly, CrossNet memories provide a capacity higher than that of “Ternary Content-Addressable Memories” with the same number of nonvolatile memory cells (e.g., memristors), and the input noise immunity of the CrossNet memories is lower.

### Neurons and Networks

Collective firing patterns of thousands of inter-connected neurons have been simulated with sophisticated computational approaches. Their monitoring requires simultaneous measurements of connectivity, synaptic strengths, and delays (Goldental et al.). Such a computational tool allows the study of recurrent neural networks that are capable of “dictating” network's connectivity and synaptic strengths. The method proposed by Goldental et al. is based on the response of neurons and depends exclusively on their recent history of stimulation. It uses a sequential chart for stimulation and recording of single neurons, in order to “mimic” a recurrent neural network with simultaneous measurements of neurons' activity. Utilization of this technique provides evidence for the emergence of spontaneous synchronous oscillations and the network's synchrony (Tang-Schomer et al.). In particular, the cooperative phenomena that include coexistence of fast γ and slow δ oscillations opens the possibility for the experimental study of large-scale networks (Goldental et al.).

### Brain Machine Interfaces

A brain machine interface (BMI) is a direct communication line between the brain and an external device. Silva reviewed the recent technological capabilities for machine learning and artificial intelligence (AI) to implement “smart” nano-brain machine interfaces (nBMI). His view consists of novel technologies that will “communicate” with the brain using approaches that allow contextual learning and adaptation to dynamic functional demands. It applies to both technologies: (i) invasive (e.g., neural prosthesis), and (ii) non-invasive (e.g., electroencephalography, EEG). Advances in computation, hardware, and software (such as algorithms that learn and adapt in a contextually dependent way) will have the ability to leverage the capabilities that nanotechnology provides to the design and functionality of nBMI.

The opportunity to optically connect/interface with the mammalian/human brain *in vivo*, has favored an unparalleled investigation of functional connectivity of brain's neuronal circuitry. Pisanello et al. reviewed the role of nanotechnology for optical-neuronal interfaces, focusing on the new devices and methods for optogenetic control of neuronal firing, and on the “detection” and “triggering of action potentials” using “optically active colloidal nanoparticles.”

Future nanotechnology will allow us to interface the cloud with a human brain. Martins et al. labeled this as a “human brain/cloud interface” (“B/CI”), based on the nano technologies referred to here as “neural nanorobotics.” Neural nanorobotics may endow a “B/CI” with “controlled” connectivity between neuronal firing and external storage and processing of data, via the direct “monitoring” of the brain's ~86 billion neurons and ~200 trillion synapses. A neural nano-robotically allowed human “B/CI” might serve as a “personalized conduit,” enabling subjects to get a direct, instantaneous access to each aspect of human knowledge.

## Author Contributions

All authors listed have made a substantial, direct and intellectual contribution to the work, and approved it for publication.

### Conflict of Interest

The authors declare that the research was conducted in the absence of any commercial or financial relationships that could be construed as a potential conflict of interest.

